# MANF Produced by MRL Mouse-Derived Mesenchymal Stem Cells Is Pro-regenerative and Protects From Osteoarthritis

**DOI:** 10.3389/fcell.2021.579951

**Published:** 2021-03-02

**Authors:** Gautier Tejedor, Patricia Luz-Crawford, Audrey Barthelaix, Karine Toupet, Sébastien Roudières, François Autelitano, Christian Jorgensen, Farida Djouad

**Affiliations:** ^1^IRMB, INSERM, University of Montpellier, Montpellier, France; ^2^Laboratorio de Inmunología Celular y Molecular, Facultad de Medicina, Universidad de los Andes, Santiago, Chile; ^3^Sanofi, Chilly-Mazarin, France; ^4^EVOTEC (France) SAS, Toulouse, France; ^5^Centre Hospitalier Universitaire de Montpellier, Montpellier, France

**Keywords:** MRL mouse, regeneration, mesenchymal stem cells, MANF, chondroprotection, osteoarthritis

## Abstract

The super healer Murphy Roths Large (MRL) mouse represents the “holy grail” of mammalian regenerative model to decipher the key mechanisms that underlies regeneration in mammals. At a time when mesenchymal stem cell (MSC)-based therapy represents the most promising approach to treat degenerative diseases such as osteoarthritis (OA), identification of key factors responsible for the regenerative potential of MSC derived from MRL mouse would be a major step forward for regenerative medicine. In the present study, we assessed and compared MSC derived from MRL (MRL MSC) and C57BL/6 (BL6 MSC) mice. First, we compare the phenotype and the differentiation potential of MRL and BL6 MSC and did not observe any difference. Then, we evaluated the proliferation and migration potential of the cells and found that while MRL MSC proliferate at a slower rate than BL6 MSC, they migrate at a significantly higher rate. This higher migration potential is mediated, in part, by MRL MSC-secreted products since MRL MSC conditioned medium that contains a complex of released factors significantly increased the migration potential of BL6 MSC. A comparative analysis of the secretome by quantitative shotgun proteomics and Western blotting revealed that MRL MSC produce and release higher levels of mesencephalic astrocyte-derived neurotrophic factor (MANF) as compared to MSC derived from BL6, BALB/c, and DBA1 mice. MANF knockdown in MRL MSC using a specific small interfering RNA (siRNA) reduced both MRL MSC migration potential in scratch wound assay and their regenerative potential in the ear punch model in BL6 mice. Finally, injection of MRL MSC silenced for MANF did not protect mice from OA development. In conclusion, our results evidence that the enhanced regenerative potential and protection from OA of MRL mice might be, in part, attributed to their MSC, an effective reservoir of MANF.

## Introduction

The super healer Murphy Roths Large (MRL) mice, is an attractive model to study tissue regeneration in mammals. Indeed, MRL mice display the extraordinary capacity to regenerate several musculoskeletal tissues such as ear wounds, injured articular cartilage and amputated digits without scarring (Clark et al., 1998; Fitzgerald et al., 2008; Ward et al., 2008; Kwiatkowski et al., 2016; Deng et al., 2019; Sinha et al., 2019). Although the mechanisms underlying MRL mice regenerative capabilities are intensively studied, the exact process responsible for tissue regeneration is not fully understood.

The cartilage regenerative potential of MRL mesenchymal stem cells (MSC) have been first tested in a model of posttraumatic arthritis. In this study, the authors assessed and compared the effect of the intra-articular injection of MSC derived from the bone marrow (BM) of MRL and C57BL/6 (BL6) mice and found that both MSC exhibit a similar protective effect (Diekman et al., 2013). In line with this study, a similar cartilage repair capacity has been described for MSC derived from the synovial of MRL and C57BL/6 mice when tested in a focal cartilage defect (Mak et al., 2016). However, although MRL MSC did not exhibit superior capacity to repair the cartilage compared to BL6 MSC, only MRL MSC were found within the defect area. More recently, extracellular vesicles produced by chondrogenic progenitor cells derived from MRL and CBA mice were tested in a mouse model of osteoarthritis induced by surgical destabilization of the medial meniscus (DMM). Extracellular vesicles derived from MRL cells had a greater anti-osteoarthritic potential than extracellular vesicles derived from CBA (Wang et al., 2020). In an inflammatory environment, MSC release factors that possess potent anti-inflammatory effects and influence cartilage matrix turnover (Van Buul et al., 2012). However, the anti-osteoarthritic activity of soluble factors released by MRL MSC have never been investigated in experimental model of osteoarthritis (OA).

Mesencephalic astrocyte-derived neurotrophic factor (MANF) is a protein evolutionarily conserved (Lindholm and Saarma, 2010) that is expressed by most if not all tissues in the body (Lindholm et al., 2008). MANF expression is induced upon various stress signals and generate a cytoprotective response in different systems (Lindahl et al., 2017). *In vivo*, MANF has been described for its role on tissue regeneration and inflammation resolution (Voutilainen et al., 2009; Chen et al., 2015; Neves et al., 2016). Indeed, in damaged retina of flies and mice, MANF expression is induced in innate immune cells, activating them and inducing an enhanced neuroprotection and tissue repair (Neves et al., 2016). MANF facilitates the differentiation and migration of neural progenitor cells and thus increasing neuroblast recruitment in stroke cortex (Tseng et al., 2018). Moreover, MANF is a systemic regulator of metabolic and immune homeostasis in young individuals. Indeed, MANF supplementation improves different hallmarks of liver aging including inflammation, hepatosteatosis and metabolic dysfunction (Sousa-Victor et al., 2019). These studies give rise to the questions of whether MANF is expressed in adult MRL mouse that have retained features of embryonic metabolism (Naviaux et al., 2009) and whether MSC derived from different mouse strains release different levels of MANF that could be correlated with their pro-regenerative or/and anti-osteoarthritic potential.

In the present study, we addressed whether the regenerative potential and the anti-osteoarthritic potential of MRL mice could be attributed to the intrinsic particularities of MSC focusing on the role of MANF.

## Materials and Methods

### MSC Isolation and Expansion

Mesenchymal stem cells (MSC) were isolated from the bone marrow (BM) of MRL/Mpj (MRL MSC) and C57BL/6 mice (BL6 MSC), expanded and phenotypically and functionally characterized after their spontaneous immortalization *in vitro* as previously described by our laboratory (Bouffi et al., 2010). The MSC were used between passages 15 and 20 for the present study.

### MSC Immunophenotype

MRL and BL6 MSC were phenotypically characterized according to the expression levels of hematopoietic and stromal cell antigens using specific antibodies against CD73, CD90, interferon (IFN)-γR1, F4/80, CD44, stem cells antigen-1 (SCA-1), CD11b, and CD45 (BD Pharmingen) and evaluated by fluorescence-activated cell sorting (FACS). Analysis was performed using the FlowJo software (BD Pharmingen).

### MSC Differentiation

Mesenchymal stem cell was induced to differentiate into chondrocytes, adipocytes and osteoblasts as previously described (Bouffi et al., 2010). Briefly, for adipogenesis, cells were seeded at 10^4^ cells/cm^2^ in complete Dulbecco’s modified Eagle’s medium (DMEM)-F12 (Invitrogen) containing 16 μM biotin, 18 μM pantothenic acid, 100 μM ascorbic acid, 5 μg/ml insulin, 0.03 μM dexamethasone, 1 μg/ml transferring, 2 ng/ml triiodothyronine (T3) and 100 nM rosiglitazone (Sigma). Formation of lipid droplets was visualized by Oil red O staining after a 1 h fixation step in 3% glutaraldehyde. For osteogenesis, cells were seeded at low confluence at 3 × 10^3^ cells/cm^2^ in proliferation media. After the cells have reached confluency, the media was replaced by osteogenic media composed of DMEM (Invitrogen) completed with 10% of fetal calf serum, 100 U/mL penicillin/streptomycin, 2 mmol/mL glutamine, 0.5 μg/ml of ascorbic acid and 3 μM NaH_2_PO_4_ (Sigma). The capacity of differentiated MSC to mineralize the extracellular matrix was assessed by Alizarin Red staining. The chondrogenic differentiation of MSC was induced using the micropellets protocol. To that end, cells were seeded at 2.5 × 10^5^ cells/well in 96-Well Polypropylene (NUNC), centrifuge at 400 *g* for 5 min and cultured in DMEM (Invitrogen) completed with 100 U/mL penicillin/streptomycin, 10 μM of sodium-pyruvate, 1.7 μM of ascorbic acid-2-phosphate, insulin*-*transferrin*-*selenium (ITS, Sigma) and 1 ng/ml of human Transforming Growth Factor β3 (hTGF-β3, R&D System). After 21 days, the pellets were fixed in paraformaldehyde (PFA) and immunohistochemistry was performed on paraffin sections.

### Chondrocyte Isolation, Expansion and Treatment

Chondrocytes were isolated from OA patients undergoing total knee replacement surgery as previously described (Maumus et al., 2013). Briefly, articular cartilage was harvested from the femoral condyles of 12 patients. Consent of donors was approved by the French Ministry of Research and Innovation (approvals DC2009-1052 and DC-2010-1185). Slices of knee cartilage were incubated within 2.5 mg/mL pronase (Sigma-Aldrich, Saint-Quentin-Fallavier, France) for 1 h and then at 37°C overnight within 2 mg/mL collagenase type II (Sigma). Digested slices were filtrated through cell strainer (70 μm) and the cell suspension was cultured in DMEM completed with 10% of fetal calf serum, 100 U/mL penicillin/streptomycin and 2 mmol/mL glutamine at the density of 25,000 cell/cm^2^ till the end of passage 1. Chondrocytes were plated at high density and cultured alone or with MANF (50 ng/mL; R&D System) and maintained for 7 days in minimal medium composed of DMEM supplemented with proline (0.35 mmol/L), ascorbic acid (0.17 mmol/L) and sodium pyruvate (1 mmol/L). Finally, the cells were collected for the analysis by RT-qPCR.

### MSC Proliferation

To evaluate the proliferation rate of MSC, the cells were culture in a proliferative medium containing DMEM supplemented with 10% of fetal calf serum, 100 U/mL penicillin/streptomycin and 2 mmol/mL glutamine during 7 days. The number of population doublings was estimated to compare the proliferation capacities of MSC populations. MSC were seeded at 5000 cells/cm^2^ and counted when 80–90% of confluency was reached. For the proliferation assessment using the PrestoBlue^TM^ assay (Life-Technologies, Courtaboeuf, France), MSC were seeded at low density (5000 cells/cm^2^) in a 6 well plates in proliferative medium. After 7 days, MSC number was evaluated.

### Proteome and Secretome Collection

Primary MSC derived from the bone marrow of BL6, BALB/c, DBA1, and MRL mice were cultured in a proliferative medium. Then, cells were washed three times with PBS prior to be cultured in a serum-free and phenol red-free DMEM medium overnight. MSC conditioned medium was collected (14 mL) and centrifuged at 3,000 × *g* for 5 min at 4°C to remove cells and cell debris and then filtered through 0.22 μm pore size membrane. Supernatants were adjusted to 0.025% with anionic acid labile surfactant I (AALS I, Progenta), concentrated at 4°C in VivaSpin 20 ultrafiltration units (#28-9323-58 MWCO: 3 kDa) and diafiltrated at 4°C against 50x volumes of 50 mM NH_4_HCO_3_ containing 0.025% AALS I. Protein concentration in the retentates was assessed using the NanoDrop 2000c Spectrophotometer (Thermo Fisher Scientific) and adjusted to 0.1 mg/mL with diafiltration buffer. Secretomes were kept frozen at −20°C until use. Cells were detached from the culture dishes by scraping into PBS on ice and lyzed by sonication in a lysis buffer containing 1% SDS in PBS supplemented with complete^TM^ mini EDTA-free Protease Inhibitor Cocktail (Roche) and Halt^TM^ Phosphatase Inhibitor Cocktail (Thermo Fisher Scientific) as well as 2500 units/ml of Benzonase^®^ endonuclease (purity grade II, Merck). Lysates were centrifuged at 20,000 × *g* for 15 min at room temperature to sediment undissolved material. Supernatants were collected and their protein content was quantified by the BCA protein assay (Pierce). Cell proteomes were kept frozen at −20°C until use.

### Proteolytic Digestion

Cell secretomes (15 μg protein) prepared from MRL and BL6 MSC were reduced in the presence of 10 mM dithiothreitol in 50 mM NH_4_HCO_3_ at 56°C for 30 min and then alkylated by adding 20 mM iodoacetamide for 30 min at room temperature in the dark. After the reduction and alkylation steps, proteins were digested with trypsin (0.75 μg) overnight at 37°C. After centrifugation, protein digests were collected, diluted twice with an equal volume of 0.2% (v/v) formic acid in H_2_O, and filtered through 0.22 μm pore size membrane prior to LC-MS/MS analysis.

### NanoLC-MS/MS Analysis, Proteins Identification, Quantification, and Statistical Analysis

Mass spectrometry (MS) analysis and data treatment was performed as previously described (Buzy, 2011; Autelitano et al., 2014). Briefly, peptide digests were analyzed on an Ultimate/Famos/Switchos suite of instruments (Dionex) connected to a hybrid LTQ Orbitrap mass spectrometer (Thermo Fisher Scientific) with the instruments setup and parameters described in the Supplementary Materials. Database searches were done using an internal MASCOT server (version 2.1, matrix Science^1^) using the UniProtKB/Swiss-Prot mouse protein knowledgebase^2^ with the search parameters as described in the Supplementary Methods. For protein quantification, raw data were processed by an in-house label-free software, DIFFTAL (DIFferential Fourrier Transform AnaLysis) as previously published (Autelitano et al., 2014; Delcourt et al., 2015) and described in the Supplementary Materials. For statistical analysis, DIFFTAL data normalizations (loess normalization at sample level and median central tendency at match set level), protein ratio (“Effect size”) and statistic *p*-value (ANOVA) calculations were performed using DanteR 0.0.1 software^3^. For biostatistics analysis of average differences in protein mean intensities (“Effect size”), between multiple replicate samples of MRL MSC and BL6 MSC, a one-way analysis of variance (ANOVA) was performed. “Effect size,” a simple way of quantifying the size of the differences between the experimental and the reference group, was calculated. A difference of 1.5 in the Effect size was considered as a significant difference.

### Bioinformatic Analysis

The identified proteins were analyzed using ProteinCenter bioinformatic tools (Proxeon Bioinformatics^4^). We made several protein sequences in one FASTA format file and submitted it to each program. SignalP (version 4.0^5^) was used to predict the presence of signal peptides in the identified proteins (D-cut-off values for SignalP-noTM networks > 0.45 or SignalP-TM networks > 0.5 as the default cut-off for signal peptide = ‘Yes’) (Petersen et al., 2011). The SecretomeP program (version 2.0^6^) was used to predict the possibility of non-classical protein secretion (SignalP signal peptide = ‘No’; and SecretomeP score > 0.5 in mammal proteins) (Bendtsen et al., 2004).

### Western Blot Analysis

Aliquots of cell proteomes or secretomes (20 μg protein) were mixed with 4x XT sample buffer (Bio Rad) containing 10% (v/v) 2-mercaptoethanol and heated at 60°C for 30 min before Western blot analysis. Proteins were resolved by SDS-PAGE on NuPAGE^®^ Novex^®^ 4 – 12% Bis-Tris gels (Bio Rad) using the NuPAGE^®^ MES SDS Running Buffer (Bio Rad) according to the manufacturer’s instructions. Proteins from SDS-PAGE were transferred to nitrocellulose membranes and stained with the BLOT-FastStain^TM^ Kit (G Biosciences) to determine transfer efficiency and reveal the protein expression profile of each sample. Blots were probed with antibodies directed against MANF (Abcam, Catalog # ab67271), growth arrest specific 6 (GAS6, R&D Systems, Catalog # MAB986), neudesin neurotrophic factor (NENF; Abcam, Catalog # ab74474), semaphorin 5A (SEMA5A, Abcam, Catalog # ab127002), or αTubulin. Immune complexes were visualized with enhanced chemiluminescence using the Amersham ECL Western blotting Detection Reagents (GE Healthcare) and X-ray films. Autoradiographs were scanned using the GS-800 Calibrated Densitometer (Bio-Rad). To quantitate the amount of protein present on the blot, signal volumes of MANF, GAS6, NENF, SEMA5A, and αTubulin were measured with Quantity One^®^ 1-D Analysis Software (Bio Rad) using volume analysis. The intensity value of each target protein band was normalized against the intensity value of αTubulin gel band used as the internal loading control for each sample.

### MSC Transfection With siRNA

Mesenchymal stem cell was transfected at subconfluence (60%) with 200 nM of control siRNA (siCTL) or the siRNA against MANF (siMANF) (Silencer Select RNAi, Thermo Fisher Scientific, Illkirsch) using oligofectamine reagent (Life Technologies, Courtaboeuf) and according to the supplier’s recommendations.

### RT-qPCR

Total RNA was isolated from each sample using RNeasy Mini Kit (Qiagen, Courtaboeuf) and the quantity and purity of the total RNA were determined by using a NanoDrop ND-1000 spectrophotometer (NanoDrop ND, Thermo Fisher Scientific). cDNA was synthesized by reverse transcribing 500 ng RNA into cDNA using the M-MLV enzyme (Thermo Fischer Scientific). Quantitative PCR was performed using the SybrGreen PCR Master Mix (Roche) and a LightCycler^®^ 480 Detection system, following manufacturer’s recommendations. Specific primers for mouse *Vimentin, Cadherin-11, N-Cadherin, E-Cadherin, ICAM-1, Integrinβ1, NCAM, MANF, RPS9* and human *TYPE 2B COLLAGEN (Col2B), AGGRECAN (AGN), LINK and SOX9* were designed using the Primer3 software (Table 1). Values were expressed as relative mRNA level of specific gene expression as obtained using the 2^–Δ^ ^*Ct*^ method, using the RPS9 expression as housekeeping gene.

**TABLE 1:** Proteins identified and quantified in MRL MSC and BL6 MSC.

**Gene name**	**Species**	**Forward sequence**	**Reverse sequence**
Vimentin	Mouse	CAG CTCACCA ACGACAAG CCG CAT	TCCTGCAAT TTCTCTCG CAGCCGCAT
Cadherin-11	Mouse	CCTTGCCTGCAT CGTCATCATTC	TTCCTCACC ACCCCCTTCAT
N-Cadherin	Mouse	TAGACGAGAGGC CTATCCATGC	CAGCAGCTTAA AGGCCCTCAT
E-Cadherin	Mouse	TCAACGATCCT GACCAGCAGT	TTGCTG CTTG GCCTCAAAA
ICAM-1	Mouse	CAATTTCTCATG CCGCACAG	AGCTGGAAGAT CGAAAGTCCG
Integrinβl	Mouse	CACGGATGCT GGGTTTCACT	TGTGCCCACT GCTGACTTAGG
NCAM	Mouse	CAGGAGTCCTT GGAATTCATCC	TGGAGAAGAC GGTGTGTCTGC
MANF	Mouse	CAGTTCCCTC TTGCCCATCC	GACACCCAGAAG CCCAAACC
RPS9	Mouse	GCTGTTGACGC TAGACGAGA	ATCTTCAGGCC CAGGATGTA
RPS9	Human	GATTACATCCTG GGCCTGAA	ATGAAGGACGG GATGTTCAC
Collagen-2	Human	CAGACGCTG GTGCTGCT	TCCTGGTTG CCGGACAT
Aggrecan	Human	TCGAGGACA GCGAGGCC	TCGAGGGTGTAG CGTGTAGAGA
LINK	Human	TTCCACAAG CACAAACTT TACACAT	GTGAAACTG AGTTTTGTATA ACCTCTCAGT
SOX9	Human	AGGTGCTCAAA GGCTACGAC	GTAATCCGGGT GGTCCTTCT

*Displayed columns include the HUGO ID, UniProtKB accession number, description, type and subcellular location (annotated by UniProtKB), maximum number of unique peptide, normalized protein intensitiy (Log2 value), average differences in protein mean intensities (Effect Size) between multiple replicate samples of MRL MSC and BL6 MSC (*N* = 2). SignalP (version 4.0, http://www.cbs.dtu.dk/services/SignalP4.0) was used to predict the presence of signal peptides in the identified proteins (D-cut-off values for SignalP-noTM networks >0.45 or SignalP-TM networks >0.5 as the default cut-off for signal peptid = ‘Yes’). The SecretomeP program (version 2.0, http://www.cbs.dtu.dk/services/SecretomeP2.0) was used to predict the possibility of nonclassical protein secretion (SignalP signal peptide = ‘No’; and SecretomeP score >0.5 in mammal proteins). Proteins annotated as “secreted” in UniProtKB are indicated with “Yes”.*

### Scratch Wound Healing

In order to evaluate the migratory potential of the cells, we used the scratch wound assay. 2.5 × 10^5^ cells were seeded in TC24 treated plates for imaging (Thermo Fischer Scientific) and the wound was performed manually once the cells adhered to the plastic. The wound closure was studied using an inverted microscope (Axiovert 200M, Zeiss) equipped with a motorized stage driven with Metamorph 7.0 software (Molecular Devices) (MRI facility). During the study, cells were maintained at 37°C with 5% of CO2. Images were taken each hour for a 24-h period. The wounded area was measured every hour using Image J Software. Area Under Curve (AUC), corresponding to the closure graph was calculated with GraphPad Prism software and normalized (percentage).

### Ear Punch Model

Ten-week-old C57Bl6 female mice were used. At day 0, we performed a reproducible ear hole with a 2 mm punch through the center of the ear. For the different groups, we use five different mice that were injected either with PBS (untreated) or 2.5 × 10^5^ MRL or BL6 MSC along the wound edge. Measurements of the ear wound area were performed at day 0 and day 44 using the ImageJ software on ear pictures.

### Collagenase-Induced Osteoarthritis Model

We carried out the collagenase-induced OA (CIOA) model as previously described (Toupet et al., 2015) and according to the guidelines and regulations of the Ethical Committee for animal experimentation of the Languedoc-Roussillon (Approval 5349-2016050918198875, CEEA-LR-12117). Briefly, 1U type VII collagenase in 5 μL saline was intra-articularly (IA) administered in the knee joint of C57BL/6 mice (10 weeks old) at day 0 and 2. Groups of 10 mice received MSC IA (2.5 × 10^5^ cells/5 μL saline), at day 7. At day 42, mice were euthanatized and paws were recovered for fixation in 4% formaldehyde and histological analysis.

### Histological Analysis

Hind paws were decalcified after 2 weeks incubation within a solution of formic acid 5% and then embedded in paraffin. Tibias were sectioned frontally as previously described (Ruiz et al., 2020) and stained with safranin O fast green staining. Quantification of the degradation of cartilage was performed using the modified Pritzker OARSI score as described (Toupet et al., 2015; Cosenza et al., 2017).

### Statistical Analysis

Results are expressed as the mean ± Standard Error of the Mean (SEM) and all experiments were performed at least three times. Generated *P*-values were obtained using Mann–Whitney unpaired *t*-test, two tails using GraphPad Prism 6 Software. Graphs show mean ± Standard SEM. *P*-values < 0.05 (^∗^), *P* < 0.01 (^∗∗^), or *P* < 0.001 (^∗∗∗^) were considered statistically significant. Analysis and graphical representation were performed using Graph-Pad Prism^TM^ software (Graphpad).

## Results

### MRL and BL6 MSC Exhibit a Similar Phenotype and Differentiation Potential

**FIGURE 1:**
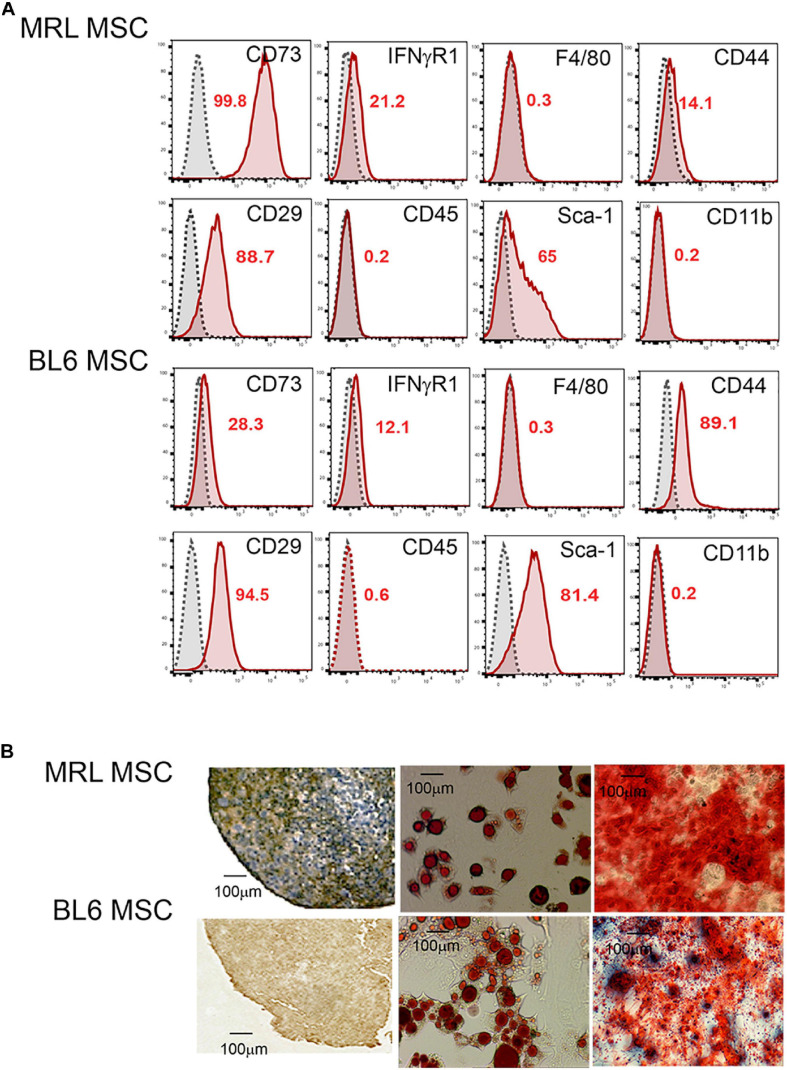
MRL MSC and BL6 MSC have the same phenotype and differentiation potential. **(A)** Phenotype of MRL and BL6 MSC was assessed by flow cytometry using different antibodies that include CD73, IFNγR1, F4/80, CD44, CD29, CD45, SCA-1, and CD11b. The percentages of positive cells are indicated in red in the flow cytometry data. **(B)** Differentiation potential of MSC. The chondrogenic differentiation of MRL and BL6 MSC was assessed using an anti-aggrecan positive staining on pellet sections. The adipogenic potential of MSC was by the visualization of lipid droplets by phase contrast microscopy at day 21 of the differentiation process. Finally, the osteogenic differentiation potential of MSC was defined by Alizarin Red S positive staining.

First, we studied the phenotype and the differentiation potential of MRL and BL6 MSC. After a long culture process in a medium containing fetal calf serum (Bouffi et al., 2010) to obtain a morphologically homogeneous MSC population with a spindle-shaped fibroblastic appearance, we phenotypically characterized the cells. Both MRL and BL6 MSC were positive for markers classically expressed by murine MSC (Bouffi et al., 2010) that include CD29, CD44, CD73, Sca-1 and they were negative for CD11b, CD45 and F4/80 (Figure 1A). Both MSC were slightly positive for IFNR1 previously demonstrated to be correlated with the immunoregulatory function of MSC (Luz-Crawford et al., 2015). Of note, the phenotypic differences observed between murine MSC derived from different strains of mice, are not related to the passage numbers but rather to do the strain of mouse they come from as we have previously reported (Bouffi et al., 2010). After induction of MSC differentiation toward the three specific lineages, both MRL and BL6 MSC gave rise to chondrocytes, adipocytes and osteoblasts as shown by the expression of aggrecan, the presence of lipid droplets in cultures and Alizarin Red S staining, respectively (Figure 1B). These results show that MRL and BL6 MSC display a similar phenotype and differentiation potential.

### MRL MSC Exhibit a Higher Migration Potential That BL6 MSC

**FIGURE 2:**
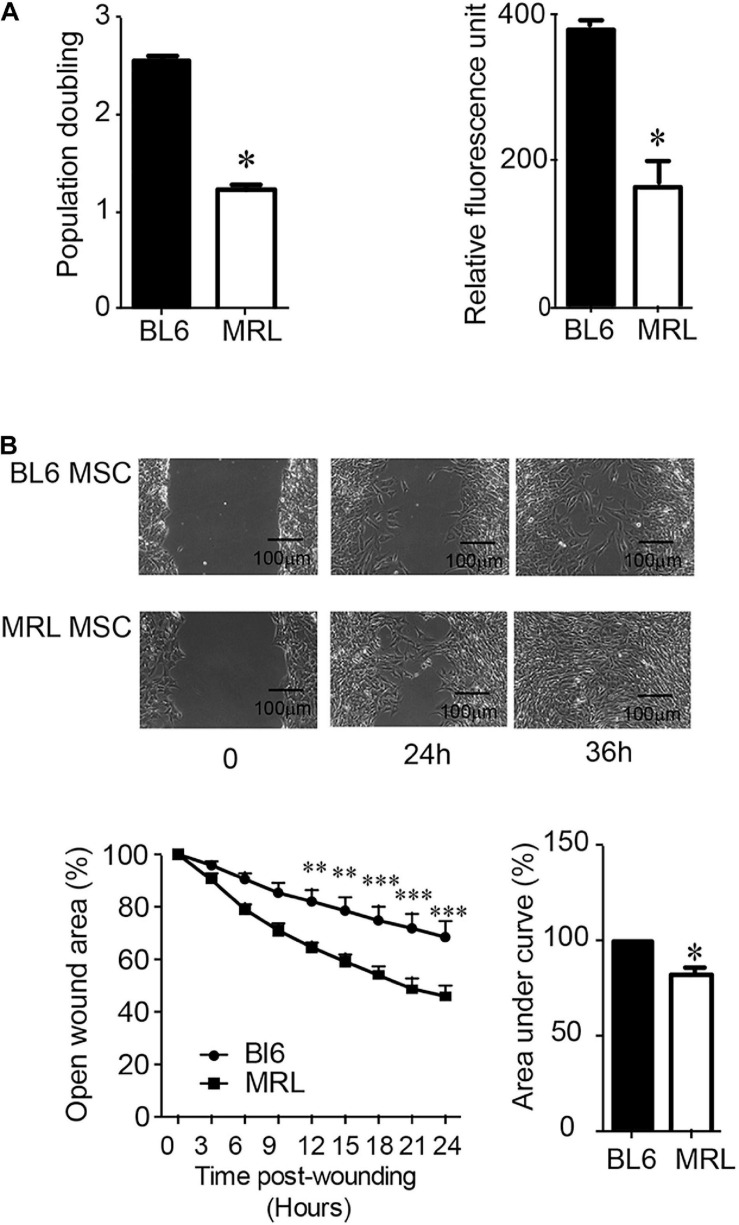
MRL MSC exhibit a lower proliferation rate and a higher migration potential than BL6 MSC. **(A)** The proliferation potential of BL6 and MRL MSC was evaluated after an overnight synchronization in absence of serum and a 7 days culture period in complete medium by either calculating the number of population doublings, i.e., the total number of times MSC has doubled from the moment we seeded (D0) them until D7 using the formula Population Doubling = 3.33 log_10_(number of MSC at D7/number of MSC plated at D0). We also used the PrestoBlue assay (Relative fluorescence unit) to evaluate the proliferation potential of the SMC. Results represented as mean ± SEM (*n* = 3 replicates). **(B)** Migration of MRL and BL6 MSC were analyzed in a scratch wound assay by evaluating the area of the wound at 0, 3, 6, 9, 12, 15, 18, 21, 24, and 36 h after wounding. Representative images from scratch wound healing assay (on the top). Measure of the open wound area (bottom). 100% correspond to the highest wound area measured at 0 h using Image J software. The area under curve (AUC) was calculated from the curve of open wound area percentage over the healing period (0–24 h). Results represented as mean ± SEM (*n* = 5 replicates). *p ≤ 0.05, **p < 0.01, ***p < 0.001.

Since a clear relationship between MSC migration and tissue repair has been established (Fu et al., 2019), we compared the migration potential of MRL and BL6 MSC. To specifically study the migration, we first compare the proliferation rate of the two cells after a synchronization step and culture within a medium containing FCS. The number of population doublings (NPD), i.e., the total number of times MSC doubled within 7 days (in a single passage) was evaluated and we found that MRL MSC showed significantly lower NPD than BL6 MSC (Figure 2A). This was confirmed using the PrestoBlue assay that defined the proliferation of MSC after 2 days of culture (Figure 2A). Then, the non-directional migration of MRL and BL6 MSC was analyzed in a scratch wound assay by evaluating the area of the wound every 3 h during 36 h post-wounding using Image J software (National Institutes of Health, Bethesda, MD, United States). Representative images from scratch wound healing assay revealed, 36 h post-wounding, a complete resurfacing of the wound for MRL MSC but not for BL6 MSC (Figure 2B). The extent of healing/migration was defined as the percentage of open wound area between the original (100% at time 0) and the residual wound area at 24 h (Figure 2B). The curve of the percentage of open wound area over the healing period (0–24 h) and the corresponding area under curve (AUC) which reflects the migration potential of the cells revealed that MRL MSC closed faster the wound than BL6 MSC (Figure 2B). Altogether these results indicate that the slow-proliferating MRL MSC display a higher migration potential than the fast-proliferating BL6 MSC.

### MRL MSC Secretome Is Responsible for Their Higher Migration Potential

**FIGURE 3:**
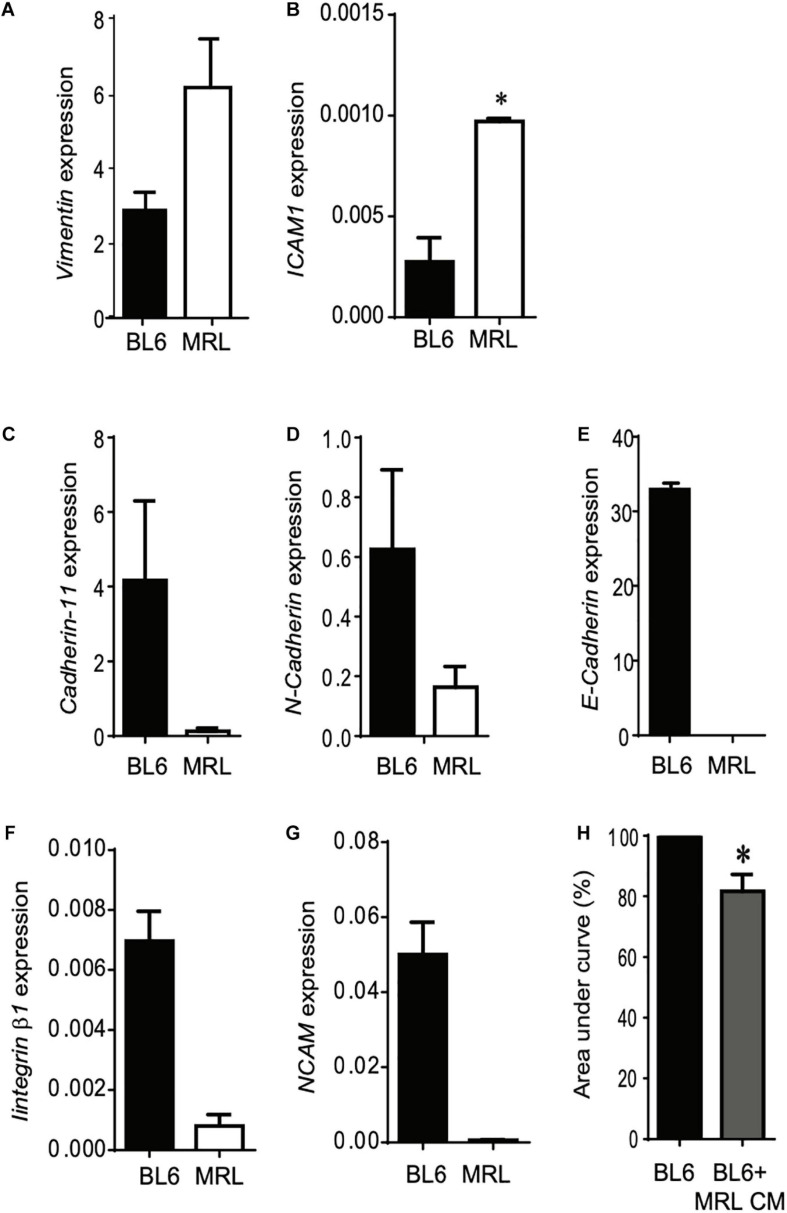
The higher migration potential of MRL MSC is mediated through the release of soluble factors. **(A–G)** RT-qPCR analysis of *Vimentin, ICAM1, Cadherin-11, N-Cadherin, E-Cadherin, Integrinβ1, and NCAM* expression levels in MRL MSC and BL6 MSC. Results represented as mean ± SEM (*n* = 4 replicates). *p ≤ 0.05. **(H)** The scratch wound was performed on BL6 MSC monolayer cultured alone or with a conditioned-medium obtained from a 24 h culture of subconfluent MRL MSC (MRL CM). The area under curve (AUC) was calculated from the curve of open wound area percentage over the healing period (0–24 h). *p ≤ 0.05.

Following their systemic injection, MSC migrate and accumulate at inflammation and injured sites (Rustad and Gurtner, 2012). The migration potential of MSC is controlled by several regulatory factors including adhesion molecules that also orchestrate their differentiation potential, immunoregulatory properties and their paracrine function in tissue repair (Ren et al., 2010; Fu et al., 2019). In this part of the study, we set out to examine how adhesive molecules could be correlated to the higher rate of migration of MRL MSC over BL6 MSC. Using RT-qPCR we studied the expression profiles of adhesion molecules in the two MSC. First, we found that *Vimentin*, whose expression is positively correlated with MSC migration (Huang et al., 2015), was highly expressed in MRL MSC as compared to BL6 MSC (Figure 3A). Similarly, ICAM-1, expressed in MSC at a low level in physiological condition and at high level in an inflammatory environment to promote MSC homing to the target and immune organs (Li et al., 2019), was expressed at a significantly higher level in MRL MSC than in BL6 MSC (Figure 3B). The other adhesion molecules studied such as *Cadherin-11* (Figure 3C), *N-Cadherin* (Figure 3D), *E-Cadherin* (Figure 3E), *Integrinβ1* (Figure 3F), and *NCAM* (Figure 3G) were expressed at a lower level in MRL MSC that in BL6 MSC. We also explored whether the higher regenerative potential of MRL MSC could be associated with soluble factors they release in culture since the regenerative potential of MSC is well-recognized to be mediated through their secretome (Menendez-Menendez et al., 2017). To that end, we cultured freshly wounded BL6 MSC with a conditioned supernatant (CM) obtained from a 24-h culture of subconfluent MRL MSC cultured in the proliferative medium (MRL CM). Scratch wounded BL6 MSC cultured with the MRL CM exhibited an enhanced capacity to resurface the wounded area as compared to BL6 MSC cultured in the proliferative medium (Figure 3H). This result suggests that the highest migration potential of MRL MSC compared to BL6 MSC is, in part, mediated through a paracrine mechanism.

### MRL MSC Produce and Release High Amount of MANF as Compared to MSC Derived From Different Mouse Strains

In order to identify factors responsible for MRL MSC migration potential, we used label-free quantitative shotgun proteomics to identify secreted proteins that are differentially expressed between MRL MSC and BL6 MSC. Secretomes of MRL MSC and BL6 MSC were analyzed based on the protein intensities measured by LC-MS/MS (*n* = 2 per cell type). A total of 904 quantifiable proteins were identified with ≥2 peptides among which 127 were annotated as “Extracellular Space” in UniProtKB/Swissprot database and 411 were predicted as secretory proteins by the *in silico* bioinformatics tools SignalP (Petersen et al., 2011) and SecretomeP (Bendtsen et al., 2004) and Swissprot protein database annotation (Supplementary Table). We identified 810 proteins differentially expressed by at least 1.5-fold between MRL MSC and BL6 MSC. Of these, 625 proteins showed increased secretion in MRL MSC, while 185 proteins showed decreased secretion compared to BL6 MSC.

**FIGURE 4:**
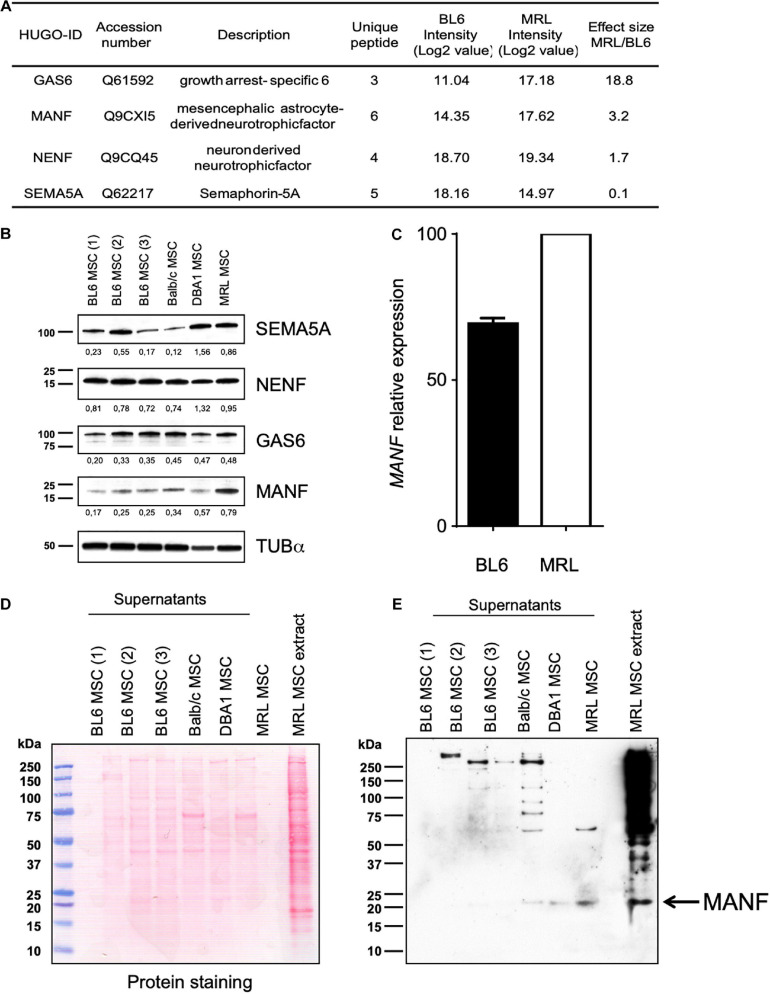
MRL MSC produced and released higher amount of MANF than BL6, BALB/c and DBA1 MSC. **(A)** Proteomic analysis of differential expression of MANF, GAS6, NENF, and SEMA5A in MRL MSC compared to BL6 MSC secretomes. Effect size indicate the standardized mean difference in protein expression level between MRL MSC and BL6 MSC. For each protein, the median intensity levels (Log 2 value) in MRL MSC and BL6 MSC are indicated. Normalized protein intensities were used to calculate the Effect size MRL/BL6. **(B)** Western blot analysis of MANF, GAS6, NENF, and SEMA5A in whole-cell extracts from BL6, BALB/c and DBA1 MSC. The intensity value of each target protein band was normalized against the intensity value of α-Tubulin gel band used as the internal loading control for each sample. **(C)** *MANF* mRNA expression level in MRL MSC and BL6 MSC. Western blot analysis of supernatants of BL6, BALB/c, and DBA1 MSC showing blotted proteins stained with Ponceau S **(D)** and the MANF protein band revealed by the anti-MANF antibody **(E)**. MRL MSC whole cell extract was used as a positive control.

Since nerve regeneration in the MRL ear wound preceded vascularization (Buckley et al., 2011), recapitulating early mammalian development and that denervation of the ear annihilated MRL mouse regenerative ability (Buckley et al., 2012), we focused our attention on proteins with neurotrophic properties showing more than 1.5-fold increased secretion in MRL MSC compared to BL6 MSC. Among these proteins, we identified GAS6, NENF, and MANF (Figure 4A). Regarding the factors showing increased secretion in BL6 MSC compared to MRL MSC, we considered one member of the semaphorin family: SEMA5A. Using Western blot method, we studied the expression profiles of MANF, NENF, GAS6 and SEMA5a in MSC from different mouse strains to identify proteins specifically highly expressed in MRL-MSC (Figure 4B). We found that MANF was the only protein among the four tested to be overexpressed in MRL MSC as compared to MSC derived from the bone marrow of BL6, BALB/c, and DBA1 mice (Figure 4B). By RT-qPCR we confirmed that MANF was overexpressed in MRL MSC as compared to BL6 MSC (Figure 4C).

Since, the MRL CM contained factors that enhanced the migration potential of BL6 MSC, we studied the differential expression profile of the candidate factors in the conditioned medium (CM) of MSC derived from MRL, BL6, BALB/c and DBA1 mice using Western blotting. We found that while MANF was not or poorly produced in the CM of BL6, BALB/c and DBA1 it was highly released in MRL MSC cultures (Figures 4D,E). These results identified MANF as a factor potentially involved in the regenerative process of MRL mice.

### MANF Is Necessary for the Regenerative Potential of MRL MSC *in vitro* and *in vivo*

**FIGURE 5:**
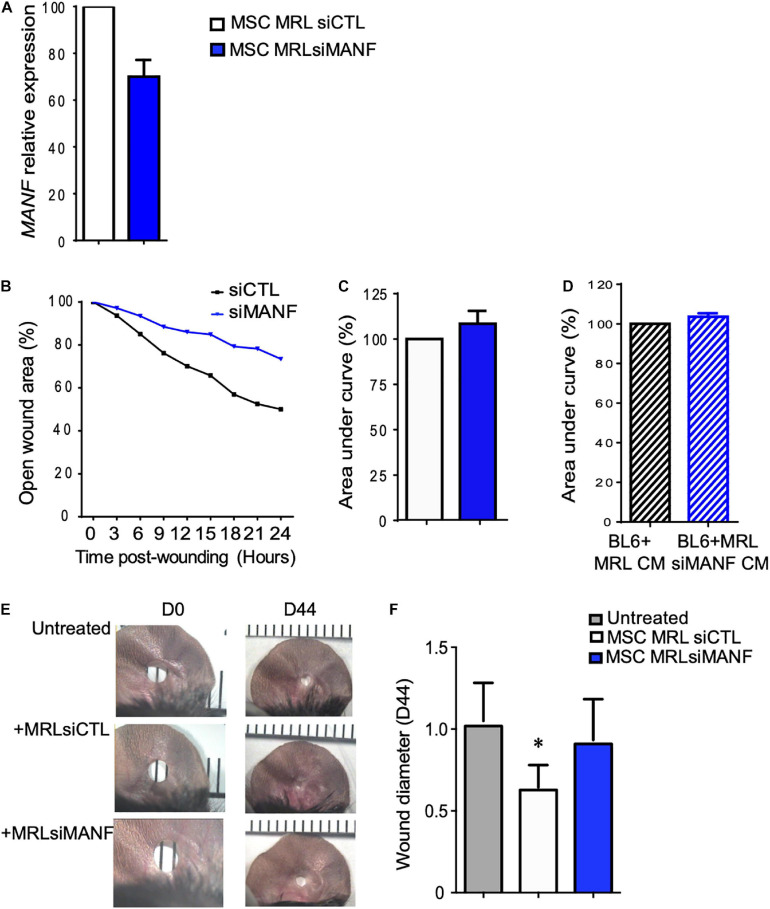
The high migration and regenerative potential of MRL MSC depend on MANF. **(A)** *MANF* mRNA expression level in MRL MSC transfected either with a control siRNA (siRNA CTL) or a siRNA against *PYCR1* (siMANF) 48 h post-transfection (mean ± SEM, from one representative experiment). **(B)** The migration potential of MRL MSC transfected with siCTL or siMANF were analyzed in a scratch wound assay by evaluating the area of the wound at 0, 3, 6, 9, 12, 15, 18, 21, and 24 h after wounding (mean ± SEM, from one representative experiment). **(C)** The area under curve (AUC) was calculated from the curve of open wound area percentage over the healing period (0–24 h). **(D)** The scratch wound was performed on BL6 MSC monolayer cultured alone or with a conditioned-medium obtained from a 24 h culture of subconfluent MRL MSC silenced for MANF (MRL siMANF CM) (mean ± SEM, *n* = 3.). **(E)** Pictures of the ear holes at day 0 and day 44 after wounding. The punch-holes in the ears of MRL mice were either untreated (untreated) or treated with MSC. MRL MSC transfected either with siCTL (MRLsiCTL) or siMANF (MRLsiMANF) were injected at the wound edges. **(F)** Quantification of the ear punch hole closure at day 44 using the image J program to define the ear punch area. Results represent the mean ± SEM. *N_*mice*_* = 10 per condition, Mann–Whitney test, two-tailed, when not indicated untreated versus MRL MSC_*siCTL*_, ^∗^*p* ≤ 0.05.

To go further, we studied the role of MANF on the regenerative potential of MRL MSC. First, we tested the role of MANF on the non-directional migration potential of MRL MSC using the scratch wound assay. To that end, we used the small interfering RNA (siRNA) approach to knock down the expression of *MANF* in MRL MSC. 48 h post-transfection (at day 0 of the scratch wound assay) of MSC with a siRNA against *MANF* (siMANF), *MANF* expression was reduced by 30% compared with the MSC transfected with the control siRNA (siCTL) (Figure 5A). The percentage of open wound area over the 24-h healing period (Figure 5B) and the corresponding AUC (Figure 5C) which reflects the migration potential of the cells revealed that *MANF* silencing reduced the capacity of MRL MSC to resurface the wound area. To define whether MANF released by MRL MSC is responsible for their migration potential, we cultured freshly wounded BL6 MSC with a conditioned supernatant (CM) obtained from a 24-h culture of subconfluent MRL MSC transfected with siMANF (MRL siMANF CM). Scratch wounded BL6 MSC cultured with the MRL siMANF CM tend to exhibit a reduced capacity to resurface the wounded area compared to BL6 MSC cultured with the control MRL CM as revealed by the higher AUC (Figure 5D). Altogether these results suggest that the high migration potential of MRL MSC, *in vitro*, partly depends on their capacity to release MANF.

The MRL mouse has been well described for its remarkable capacity for cartilaginous wound closure and regeneration two decades ago (Clark et al., 1998). Indeed, 2 mm punch wounds made into MRL/MpJ mice ears closed with regeneration after 30 days, whereas they did not close in the C57BL/6 mice. Herein, we tested whether the administration of MRL MSC could induce the regeneration in BL6 mice and whether this relies on MANF production. To that end, a 2 mm punch wounds was made into BL6 mice. Wounded mice were either untreated or treated with MSC (MRL siCTL) injected along the wound edge. Measurements of the ear punch wound area at day 44 revealed that MRL MSC induced a regenerative process leading to a significant decrease of the wound size, 40% reduction in area, as compared to the untreated mice (Figures 5E,F). Then, we assessed, whether this regenerative process mediated by MRL MSC was associated with their high expression level of *MANF*. The ear punch wound area of BL6 mice treated with MRL MSC deficient for *MANF* (MRL MSC siMANF) did not show any difference with the untreated mice (Figures 5E,F). Overall, this finding suggests that the *in vivo* regenerative potential of MRL MSC in BL6 mice depends, in part, on *MANF* expression level.

### MANF Is Necessary for the Anti-osteoarthritic Properties of MRL MSC

Mesenchymal stem cell protect chondrocytes from degeneration associated with OA and protect mice from OA development (Maumus et al., 2013; Toupet et al., 2015; Cosenza et al., 2017; Ruiz et al., 2020). Since MRL mice possess an intrinsic ability to regenerate articular cartilage (Fitzgerald et al., 2008), we wondered whether MANF highly produced by MRL MSC could protect chondrocyte from a loss of anabolic markers *in vitro* and mice from OA development *in vivo*.

**FIGURE 6:**
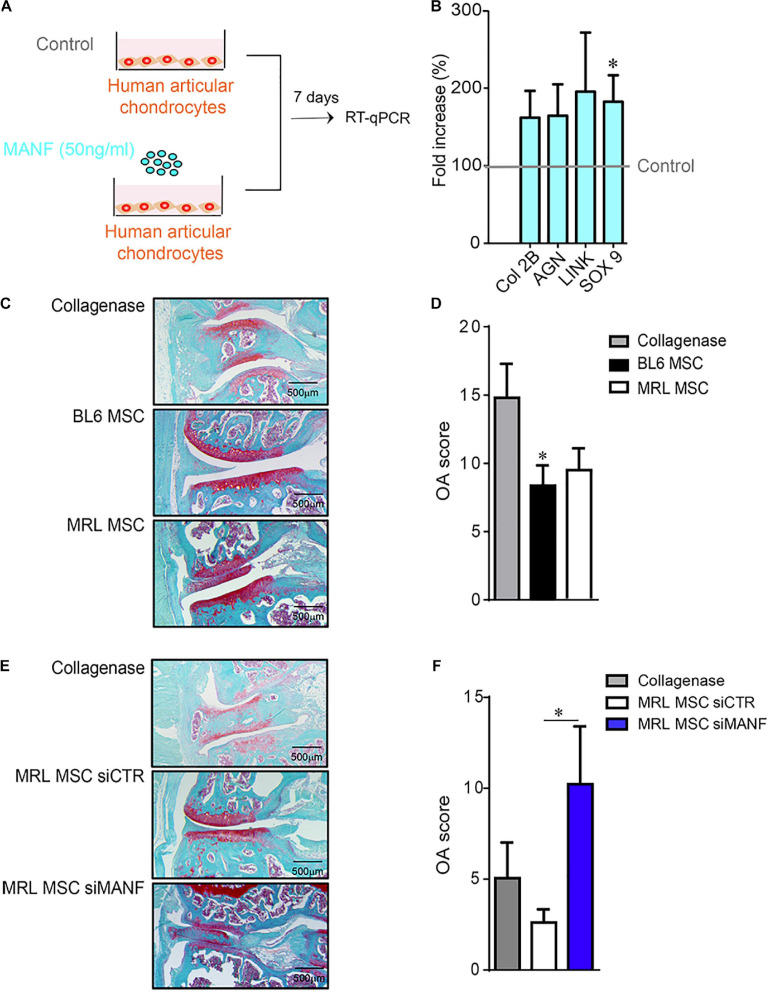
MANF protects chondrocyte from a OA-like phenotype and mice from osteoarthritis. **(A)** Scheme showing the treatment protocol of human primary chondrocytes with MANF (50 ng/mL) during 7 days. **(B)** RT-qPCR analysis of chondrocyte anabolic markers such as *Col2B, AGN, LINK*, and *SOX9* expressed in human primary chondrocytes cultured in minimal medium or minimal medium (Control) containing MANF during 7 days. Results are expressed as fold change of gene expression compared to chondrocytes alone (Control) [mean ± SEM (*n* = 12 biological replicates)]. *p ≤ 0.05. **(C)** Histological images of CIOA mice not treated (Collagenase) or treated with BL6 MSC or MRL MSC. **(D)** OA score of histological sections of knee joints of the mice described in **(C)**. **(C)** (*n* = 15/group). **(E)** Histological images of CIOA mice not treated (Collagenase) or treated with MRL MSC transfected with a siCTL (MRL MSC siCTL) or a siMANF (MRL MSC siMANF). **(F)** OA score of histological sections of knee joints of the mice described in **(E)**. Results are expressed as the mean ± SEM; *p < 0.05 (Mann–Whitney test; *n* = 10 mice/group).

In order to determine the effect of MANF on human primary chondrocytes, a culture assay was designed. To that end, we used human primary chondrocytes that were isolated and cultured in proliferative medium containing 10% FCS. Then, chondrocytes were placed in a minimal medium to avoid FCS side effects and culture alone or with recombinant human MANF (50 ng/mL) during 7 days (Figure 6A). Then, we evaluated the phenotype of chondrocytes in the culture assay and found that while MANF tend to increase the expression of chondrocyte markers such as *Col 2B*, *Aggrecan* (*AGN*) and *LINK* its significantly increased the expression of *SOX9* (Figure 6B). Altogether, these data demonstrate a potent role of MANF in the protection of chondrocytes from the loss of mature chondrocyte phenotype which are characteristics of OA.

Then, we evaluated *in vivo* the effect of IA injection of BL6 and MRL MSC in CIOA mice (Toupet et al., 2015). As expected, histological analysis showed that the OA score was significantly lower in CIOA mice treated with BL6 MSC compared with untreated mice (Figures 6C,D). Of note, we did not observe any significant difference between the OA score of mice treated with either BL6 or MRL MSC. Conversely, the OA score was significantly higher in mice treated with MRL MSC silenced for *MANF* (MRL MSC siMANF) as compared to mice treated with MRL MSC (Figures 6E,F). Overall, these results indicate that MANF highly produced by MRL MSC protects chondrocytes from a loss of anabolic markers that characterizes OA-like chondrocytes and contributes to their therapeutic effect in CIOA mice.

## Discussion

This study provides the first evidence that MRL MSC induce ear hole regeneration and protect mice from osteoarthritis through their high capacity to release MANF.

First, we showed that the slow-proliferating MRL MSC display a higher migration potential than the fast-proliferating BL6 MSC. This is in line with the established relationship between MSC migration and tissue repair (Fu et al., 2019). Due to their paracrine ability, migration, adhesion and homing potential, MSC have been intensively studied in the field of regenerative medicine. Thus, it is tempting to believe that the identification of key factors for MRL MSC migration and regeneration potential will be of high interest for novel therapy of degenerative diseases. Thus, due to their enhanced migration potential mediated, in part, through the release of paracrine factors, we focused on the secretome of MRL MSC. We found that MANF was specifically highly produced by MRL MSC as compared to MSC derived from other mouse strains. The role of MANF in tissue regeneration has already been described. Indeed, in mice and flies, in response to retina damages retina of flies and mice, MANF was induced in innate immune cells. MANF activated innate immune cells, enhanced cytoprotection and improved tissue repair (Neves et al., 2016). In this study, MANF was shown to be necessary and sufficient to induce the *Drosophila* homolog of the mammalian anti-inflammatory macrophage marker *arginase1* in hemocytes, suggesting an association between MANF expression, resolution of inflammation and tissue repair in the retina (Neves et al., 2016).

Osteoarthritis (OA) is an inflammatory and degenerative joint disease, which mainly affects the articular cartilage and results in pain and impaired movement. It is now acknowledged that OA is a heterogeneous disease with multiple clinical phenotypes and that inflammation contributes significantly to cartilage and bone alterations during OA. However, conventional anti-inflammatory therapies have been disappointing so far for the treatment of OA. Thus the identification of a molecule that would not only control inflammation but also promote MSC migration and regeneration would be of high interest to treat most if not all degenerative diseases characterized by two main features that are inflammation and tissue degradation. Here, we demonstrate that the high migration potential of MRL MSC, *in vitro*, and the regenerative potential of MRL MSC in ear punched BL6 mice depends on *MANF*. Thus in addition to their anti-inflammatory effects described in the context of damaged retina, here, we provide the first evidence that MANF can control the migration and the regenerative properties of MSC. This is in line with the study performed in stroke cortex and that describes the neuroregenerative activity of MANF through its capacity to favor differentiation and migration of neural progenitor cells (Tseng et al., 2018). Overexpressed MANF induced STAT3 activation during subventricular zone cell migration (Tseng et al., 2018). In MSC, STAT3 silencing inhibited their migration and invasion ability while IL22 also promoted MSC migration and invasion through STAT3 signaling (Cui et al., 2019).

We also described the pro-regenerative role or MANF in the ear punch model. Indeed, while MRL MSC induced a regenerative process, MRL MSC deficient for *MANF* did not. The tissue repair potential of MANF was proposed to function through a synergistic activity as a suppressor of inflammation and apoptosis (Sousa-Victor et al., 2018). This model is in agreement with our recent findings showing the anti-apoptotic effect of MANF on human primary chondrocytes treated with camptothecin (data not shown).

Finally, we showed that MANF highly produced by MRL MSC contributes to their capacity to tend to reduce the OA score. However, although we observed a significant exacerbation of the OA score in mice treated with MRL MSC deficient for *MANF* as compared to naïve MRL MSC, we did not show an enhanced therapeutic effect of MRL MSC compared to BL6 MSC in CIOA mice. This result underlines that other factors than MANF are involved in the anti-osteoarthritic properties of MSC. Regarding the *in vivo* beneficial effect of MSC, it is still not clear whether it could be due to the immunoregulatory properties of MSC or to tissue-protective and pro-regenerative properties. Further studies should be performed to define the mechanisms underlying the anti-osteoarthritic properties of MSC.

## Conclusion

Our findings demonstrate that the enhanced regenerative potential of MRL MSC as well as their capacity to tend to reduce OA is attributed, in part, to their capacity to release high amount of MANF.

## Data Availability Statement

The original contributions presented in the study are included in the article/Supplementary Material, further inquiries can be directed to the corresponding author/s.

## Ethics Statement

The animal study was reviewed and approved by 5349-2016050918198875, CEEA-LR-12117.

## Author Contributions

FD designed the project and the experiments with the input of PL-C, FA, and CJ. GT, PL-C, AB, KT, and SR performed the experiments and analyzed the results. FD wrote the manuscript with the input of PL-C, FA, and CJ. All authors contributed to the article and approved the submitted version.

## Conflict of Interest

FA was employed by the company Evotec France (SAS). SR was employed by the company Sanofi. The remaining authors declare that the research was conducted in the absence of any commercial or financial relationships that could be construed as a potential conflict of interest.

## Footnotes

1 http://www.matrixscience.com/

2 http://www.uniprot.org/

3 http://omics.pnl.gov/software/

4 http://www.cbs.dtu.dk/services

5 http://www.cbs.dtu.dk/services/SignalP4.0

6 http://www.cbs.dtu.dk/services/SecretomeP2.0

## References

[B1] Buzy A. (2011). *DIFFTAL : A label-free approach for absolute quantification of proteins in a complex mixture. presented at the annual meeting “6ème Journée de Spectrométrie de Masse en Midi-Pyrénées”, Toulouse, France, 13 December 2011.*

[B2] Autelitano F. Loyaux D. Roudieres S. Deon C. Guette F. Fabre P. (2014). Identification of novel tumor-associated cell surface sialoglycoproteins in human glioblastoma tumors using quantitative proteomics. *PLoS One* 9:e110316. 10.1371/journal.pone.0110316 25360666 PMC4216004

[B3] Bendtsen J. D. Jensen L. J. Blom N. Von Heijne G. Brunak S. (2004). Feature-based prediction of non-classical and leaderless protein secretion. *Protein Eng Des Sel* 17 349–356. 10.1093/protein/gzh037 15115854

[B4] Bouffi C. Bony C. Courties G. Jorgensen C. Noel D. (2010). IL-6-dependent PGE2 secretion by mesenchymal stem cells inhibits local inflammation in experimental arthritis. *PLoS One* 5:e14247. 10.1371/journal.pone.0014247 21151872 PMC2998425

[B5] Buckley G. Metcalfe A. D. Ferguson M. W. (2011). Peripheral nerve regeneration in the MRL/MpJ ear wound model. *J Anat* 218 163–172. 10.1111/j.1469-7580.2010.01313.x 20950365 PMC3042750

[B6] Buckley G. Wong J. Metcalfe A. D. Ferguson M. W. (2012). Denervation affects regenerative responses in MRL/MpJ and repair in C57BL/6 ear wounds. *J Anat* 220 3–12. 10.1111/j.1469-7580.2011.01452.x 22066944 PMC3248659

[B7] Chen L. Feng L. Wang X. Du J. Chen Y. Yang W. (2015). Mesencephalic astrocyte-derived neurotrophic factor is involved in inflammation by negatively regulating the NF-kappaB pathway. *Sci Rep* 5 8133. 10.1038/srep08133 PMC4313098 25640174

[B8] Clark L. D. Clark R. K. Heber-Katz E. (1998). A new murine model for mammalian wound repair and regeneration. *Clin Immunol Immunopathol* 88 35–45. 10.1006/clin.1998.4519 9683548

[B9] Cosenza S. Ruiz M. Toupet K. Jorgensen C. Noel D. (2017). Mesenchymal stem cells derived exosomes and microparticles protect cartilage and bone from degradation in osteoarthritis. *Sci Rep* 7 16214. 10.1038/s41598-017-15376-8 PMC5701135 29176667

[B10] Cui X. Jing X. Yi Q. Xiang Z. Tian J. Tan B. (2019). IL22 furthers malignant transformation of rat mesenchymal stem cells, possibly in association with IL22RA1/STAT3 signaling. *Oncol Rep* 41 2148–2158. 30816520 10.3892/or.2019.7007 PMC6412447

[B11] Delcourt N. Quevedo C. Nonne C. Fons P. O’brien D. Loyaux D. (2015). Targeted identification of sialoglycoproteins in hypoxic endothelial cells and validation in zebrafish reveal roles for proteins in angiogenesis. *J Biol Chem* 290 3405–3417. 10.1074/jbc.m114.618611 25384978 PMC4319010

[B12] Deng Z. Gao X. Sun X. Amra S. Lu A. Cui Y. (2019). Characterization of articular cartilage homeostasis and the mechanism of superior cartilage regeneration of MRL/MpJ mice. *FASEB J* 2019 fj201802132RR. 10.1096/fj.201802132RR 31042406

[B13] Diekman B. O. Wu C. L. Louer C. R. Furman B. D. Huebner J. L. Kraus V. B. (2013). Intra-articular delivery of purified mesenchymal stem cells from C57BL/6 or MRL/MpJ superhealer mice prevents posttraumatic arthritis. *Cell Transplant* 22 1395–1408. 10.3727/096368912x653264 22889498 PMC3891895

[B14] Fitzgerald J. Rich C. Burkhardt D. Allen J. Herzka A. S. Little C. B. (2008). Evidence for articular cartilage regeneration in MRL/MpJ mice. *Osteoarthritis Cartilage* 16 1319–1326. 10.1016/j.joca.2008.03.014 18455447

[B15] Fu X. Liu G. Halim A. Ju Y. Luo Q. Song A. G. (2019). Mesenchymal Stem Cell Migration and Tissue Repair. *Cells* 2019 8. 10.3390/cells8080784 PMC6721499 31357692

[B16] Huang L. Niu C. Willard B. Zhao W. Liu L. He W. (2015). Proteomic analysis of porcine mesenchymal stem cells derived from bone marrow and umbilical cord: implication of the proteins involved in the higher migration capability of bone marrow mesenchymal stem cells. *Stem Cell Res Ther* 6 77. 10.1186/s13287-015-0061-x PMC4425931 25889491

[B17] Kwiatkowski A. Piatkowski M. Chen M. Kan L. Meng Q. Fan H. (2016). Superior angiogenesis facilitates digit regrowth in MRL/MpJ mice compared to C57BL/6 mice. *Biochem Biophys Res Commun* 473 907–912. 10.1016/j.bbrc.2016.03.149 27040769

[B18] Li X. Wang Q. Ding L. Wang Y. X. Zhao Z. D. Mao N. (2019). Intercellular adhesion molecule-1 enhances the therapeutic effects of MSCs in a dextran sulfate sodium-induced colitis models by promoting MSCs homing to murine colons and spleens. *Stem Cell Res Ther* 10 267. 10.1186/s13287-019-1384-9 PMC6708236 31443680

[B19] Lindahl M. Saarma M. Lindholm P. (2017). Unconventional neurotrophic factors CDNF and MANF: Structure, physiological functions and therapeutic potential. *Neurobiol Dis* 97 90–102. 10.1016/j.nbd.2016.07.009 27425895

[B20] Lindholm P. Peranen J. Andressoo J. O. Kalkkinen N. Kokaia Z. Lindvall O. (2008). MANF is widely expressed in mammalian tissues and differently regulated after ischemic and epileptic insults in rodent brain. *Mol Cell Neurosci* 39 356–371. 10.1016/j.mcn.2008.07.016 18718866

[B21] Lindholm P. Saarma M. (2010). Novel CDNF/MANF family of neurotrophic factors. *Dev Neurobiol* 70 360–371. 20186704 10.1002/dneu.20760

[B22] Luz-Crawford P. Torres M. J. Noel D. Fernandez A. Toupet K. Alcayaga-Miranda F. (2015). The Immunosuppressive Signature of Menstrual Blood Mesenchymal Stem Cells Entails Opposite Effects on Experimental Arthritis and Graft Versus Host Diseases. *Stem Cells.* 34 456–469. 26528946 10.1002/stem.2244

[B23] Mak J. Jablonski C. L. Leonard C. A. Dunn J. F. Raharjo E. Matyas J. R. (2016). Intra-articular injection of synovial mesenchymal stem cells improves cartilage repair in a mouse injury model. *Sci Rep* 6 23076. 10.1038/srep23076 PMC4794799 26983696

[B24] Maumus M. Manferdini C. Toupet K. Peyrafitte J. A. Ferreira R. Facchini A. (2013). Adipose mesenchymal stem cells protect chondrocytes from degeneration associated with osteoarthritis. *Stem Cell Res* 11 834–844. 10.1016/j.scr.2013.05.008 23811540

[B25] Menendez-Menendez Y. Otero-Hernandez J. Vega J. A. Perez-Basterrechea M. Perez-Lopez S. Alvarez-Viejo M. (2017). The role of bone marrow mononuclear cell-conditioned medium in the proliferation and migration of human dermal fibroblasts. *Cell Mol Biol Lett* 22 29. 10.1186/s11658-017-0055-z PMC5735620 29270201

[B26] Naviaux R. K. Le T. P. Bedelbaeva K. Leferovich J. Gourevitch D. Sachadyn P. (2009). Retained features of embryonic metabolism in the adult MRL mouse. *Mol Genet Metab* 96 133–144. 10.1016/j.ymgme.2008.11.164 19131261 PMC3646557

[B27] Neves J. Zhu J. Sousa-Victor P. Konjikusic M. Riley R. Chew S. (2016). Immune modulation by MANF promotes tissue repair and regenerative success in the retina. *Science* 353 aaf3646. 10.1126/science.aaf3646 27365452 PMC5270511

[B28] Petersen T. N. Brunak S. Von Heijne G. Nielsen H. (2011). SignalP 4.0: discriminating signal peptides from transmembrane regions. *Nat Methods* 8 785–786. 10.1038/nmeth.1701 21959131

[B29] Ren G. Zhao X. Zhang L. Zhang J. L’huillier A. Ling W. (2010). Inflammatory cytokine-induced intercellular adhesion molecule-1 and vascular cell adhesion molecule-1 in mesenchymal stem cells are critical for immunosuppression. *J Immunol* 184 2321–2328. 10.4049/jimmunol.0902023 20130212 PMC2881946

[B30] Ruiz M. Toupet K. Maumus M. Rozier P. Jorgensen C. Noel D. (2020). TGFBI secreted by mesenchymal stromal cells ameliorates osteoarthritis and is detected in extracellular vesicles. *Biomaterials* 226 119544. 10.1016/j.biomaterials.2019.119544 31648137

[B31] Rustad K. C. Gurtner G. C. (2012). Mesenchymal Stem Cells Home to Sites of Injury and Inflammation. *Adv Wound Care* 1 147–152. 10.1089/wound.2011.0314 24527296 PMC3623614

[B32] Sinha K. M. Tseng C. Guo P. Lu A. Pan H. Gao X. (2019). Hypoxia-inducible factor 1alpha (HIF-1alpha) is a major determinant in the enhanced function of muscle-derived progenitors from MRL/MpJ mice. *FASEB J* 33 8321–8334. 10.1096/fj.201801794r 30970214 PMC6593884

[B33] Sousa-Victor P. Jasper H. Neves J. (2018). Trophic Factors in Inflammation and Regeneration: The Role of MANF and CDNF. *Front Physiol* 9:1629. 10.3389/fphys.2018.01629 PMC6255971 30515104

[B34] Sousa-Victor P. Neves J. Cedron-Craft W. Ventura P. B. Liao C. Y. Riley R. R. (2019). MANF regulates metabolic and immune homeostasis in ageing and protects against liver damage. *Nat Metab* 1 276–290. 10.1038/s42255-018-0023-6 31489403 PMC6727652

[B35] Toupet K. Maumus M. Luz-Crawford P. Lombardo E. Lopez-Belmonte J. Van Lent P. (2015). Survival and biodistribution of xenogenic adipose mesenchymal stem cells is not affected by the degree of inflammation in arthritis. *PLoS One* 10:e0114962. 10.1371/journal.pone.0114962 25559623 PMC4283953

[B36] Tseng K. Y. Anttila J. E. Khodosevich K. Tuominen R. K. Lindahl M. Domanskyi A. (2018). MANF Promotes Differentiation and Migration of Neural Progenitor Cells with Potential Neural Regenerative Effects in Stroke. *Mol Ther* 26 238–255. 10.1016/j.ymthe.2017.09.019 29050872 PMC5763030

[B37] Van Buul G. M. Villafuertes E. Bos P. K. Waarsing J. H. Kops N. Narcisi R. (2012). Mesenchymal stem cells secrete factors that inhibit inflammatory processes in short-term osteoarthritic synovium and cartilage explant culture. *Osteoarthritis Cartilage* 20 1186–1196. 10.1016/j.joca.2012.06.003 22771777

[B38] Voutilainen M. H. Back S. Porsti E. Toppinen L. Lindgren L. Lindholm P. (2009). Mesencephalic astrocyte-derived neurotrophic factor is neurorestorative in rat model of Parkinson’s disease. *J Neurosci* 29 9651–9659. 10.1523/jneurosci.0833-09.2009 19641128 PMC6666534

[B39] Wang R. Jiang W. Zhang L. Xie S. Zhang S. Yuan S. (2020). Intra-articular delivery of extracellular vesicles secreted by chondrogenic progenitor cells from MRL/MpJ superhealer mice enhances articular cartilage repair in a mouse injury model. *Stem Cell Res Ther* 11 93. 10.1186/s13287-020-01594-x PMC7052980 32122385

[B40] Ward B. D. Furman B. D. Huebner J. L. Kraus V. B. Guilak F. Olson S. A. (2008). Absence of posttraumatic arthritis following intraarticular fracture in the MRL/MpJ mouse. *Arthritis Rheum* 58 744–753. 10.1002/art.23288 18311808

