# Supplementary material for: MANF Produced by MRL Mouse-Derived Mesenchymal Stem Cells Is Pro-regenerative and Protects From Osteoarthritis
Source: Front Cell Dev Biol. 2021 Mar 2;9:579951. doi: 10.3389/fcell.2021.579951 (PMC7960785; doi:10.3389/fcell.2021.579951)
Supplement: Supplementary file 1 [file Data_Sheet_1.PDF]

MANF produced by MRL mouse-derived mesenchymal stem cells is pro-regenerative and protects from osteoarthritis

Authors:

Gautier Tejedor[1], Patricia Luz-Crawford[3], Audrey Barthelaix[1], Karine Toupet[1], Sébastien Roudières[4], François Autelitano[5], Christian Jorgensen[1,2], Farida Djouad[1]

Addresses:

[1]IRMB, Univ Montpellier, INSERM, Montpellier, France ; [2]CHU Montpellier, Montpellier, F-34295 France; [3]Laboratorio de Inmunología Celular y Molecular, Facultad de Medicina, Universidad de los Andes, Santiago, Chile ; [4]SANOFI, Chilly-Mazarin, France; [5]EVOTEC (France) SAS, Toulouse, France

Key words

MRL mouse, regeneration, mesenchymal stem cells, MANF, chondroprotection, osteoarthritis

Corresponding author:

Farida Djouad, Inserm U 1183, IRMB, Hôpital Saint-Eloi, 80 avenue Augustin Fliche,

34295 Montpellier cedex 5, France. Tel: 33 (0) 4 67 33 04 75 – Fax: 33 (0) 4 67 33 01 13 –

E-mail: farida.djouad@inserm.fr

Supplementary materials

NanoLC-MS/MS analysis

LC-MS/MS experiments were performed on an Ultimate/Famos/Switchos suite of instruments (Dionex) connected to a hybrid LTQ Orbitrap mass spectrometer (Thermo Fisher Scientific) equipped with a nanoelectrospray source. Tryptic digests were loaded onto a trap column (100 Å C18 Pepmap, Dionex, 5 mm × 300 μm,) and washed with 0.2% formic acid at 30 μL/min using the Switchos pump for 5 min. Peptides were then eluted on a C18 reverse-phase nanoflow column (100 Å C18 Pepmap, Dionex, 150 mm × 75 μm) with a linear gradient of 5 – 40% solvent B ($H_2O/CH_3CN/HCOOH$, 10:90:0.2, by vol.) for 125 min, 40-90% solvent B for 20 min, and 90% solvent B for 5 min, at a flow rate of 200 nL/min. The mass spectrometer was operated in the data-dependent mode to automatically switch between MS and MS/MS acquisition. Survey full scan MS spectra (from m/z 300 – 1700) were acquired in the Orbitrap with a resolution of 60,000 at m/z 400. The AGC was set to 1×10^6 with a maximum injection time of 500 ms. The most intense ions (up to 5) were then isolated for fragmentation in the LTQ linear ion trap using a normalized collision energy of 35% at the default activation q of 0.25 with an AGC settings of 1×10^5 and a maximum injection time of 100 ms. The dynamic exclusion time window was set to 900 s. Samples were injected in triplicate. All m/z selected for MS/MS during the first LC-MS/MS experiment were excluded of MS/MS process for the next LC-MS/MS run (generation of a reject mass list with a 10 ppm m/z window and a 10 min retention time window, see LC-MS/MS data processing). The third run was then performed with a reject mass list generated from the 1st and 2nd LC-MS/MS experiments. Ion selection threshold was set to 80,000 counts for the 1st LC-MS/MS run and to 40,000 and 20,000 for the 2nd and 3rd LC-MS/MS experiments, respectively.

LC-MS/MS data processing

LC-MS/MS data, acquired using the Xcalibur software (version 2.07, Thermo-Fisher Scientific), were processed using a home-made Visual Basic program software developed using XRawfile libraries distributed by Thermo-Fisher Scientific. This program generates 4 different files. The first one corresponds to a MS/MS peak list (MGF file) which is used for database searching. The MGF file contains the exact parent mass and the retention time (RT) associated with each LTQ-MS/MS spectrum. The exact parent mass is the ^{12}C isotope ion mass of the most intense isotopic pattern detected on the high resolution Orbitrap MS parallel scan and included in the LTQ-MS/MS selection window. The RT is issued from the LTQ-MS/MS scan. The second file is a MS/MS log file which reports, for each acquired MS/MS, the scan number, the ^{12}C isotope exact mass, the RT and the parent filter (LTQ selection window). The third file corresponds to the conversion of the high resolution MS raw data file into a "csv" format file which will be used for quantitative analysis. The last file is an exclude list text file which contains the ^{12}C isotope precursor ion mass with the corresponding start/end exclude RT 10 min window. The Xcalibur software uses this exclude list as the reject mass list (specifies which parent ions cannot trigger a dependent scan) during the 2nd and 3rd LC-MS/MS experiments (see nanoLC-MS/MS analysis).

Database searching

Database searches were done using our internal MASCOT server (version 2.1, matrix Science; http://www.matrixscience.com/) using the Swiss-Prot mouse database. The search parameters used for post-translational modifications were a fixed modification of +57.02146 Da on cysteine residues (carboxyamidomethylation) and dynamic modifications of +15.99491 on methionine residues (oxidation), of +42.010565 on protein N-terminal residues (N-terminal acetylation) and -17.026549 on N-terminal glutamine residues (N-Pyroglu). The precursor mass

tolerance was set to 5 ppm and the fragment ion tolerance was set to 0.5 Da. The number of missed cleavage sites for trypsin was set to 3. Mascot result files (".dat" files) were imported into Scaffold software (http://www.proteomesoftware.com/). Queries were also used for XTandem parallel Database Search. The compiled results of both database searches were exported.

Quantitative analysis

Relative quantification was performed using in-house software, DIFFTAL (DIFferential Fourrier Transform AnaLysis) [1]. DIFFTAL is a set of software tools developed in Sanofi under MatLab environment (http://www.mathworks.com/) for label-free differential analysis of complex proteomic mixture dedicated to LTQ/Orbitrap data. DIFFTAL runs in 3 main steps: (1) Feature detection, (2) MS matching and (3) MS/MS matching.

Step1, Feature detection. Each LC/MS file is treated independently for feature detection. The signal apparition is detected scan by scan by analysing the evolution of the average signal of 3 consecutive scans. Feature detection is achieved using the peptide isotopic patterns calculated with "Averagine" algorithm (*96*). In case of overlapped peptide signals, the solution is a linear combination of theoretical patterns which minimizes the distance with the detected signal. At the end of the process, a matrix of the features detected in the 3D space (m/z, RT and intensity) is stored. This matrix contains links to retrieve the corresponding processed signals, which are stored in a temporary data bank.

Step2, MS matching. All LC-MS data are matched together using a progressive alignment procedure. The most intense detected features are first matched in agreement with m/z and RT precision windows defined by the user. All peptides are then used to compute a specific RT alignment model. A definitive RT window is calculated according to the dispersion observed

between real and calculated RTs. Finally, every remaining unmatched m/z is checked by going back to the processed signal stored during the feature detection step.

Step3, MS/MS matching. MS/MS Spectrum reports exported from Scaffold are matched with the matrix of detected features using the corresponding acquisition MS/MS log files (see LC-MS/MS data processing). This matching requires starting and ending time points of each feature. Indeed, the RT feature is the time at the maximum intensity of the observed MS signal, whereas the MS/MS spectrum is recorded at any time during the peptide elution. In case of ambiguity, the comparison between the exact isotopic profile calculated from the MS/MS sequence and the detected signal at the feature RT is used for sorting. Because about 5 – 10% of the identified peptides are lost during the MS/MS matching process, another routine has been introduced in the software. This routine quantifies only the MS/MS identified peptides according to the following scheme: the time profiles of the 2 major isotopes of each identified peptide are computed in a small time window where the MS/MS spectrum is recorded. Only the co-eluted signals of these 2 isotopes are analysed to determine the peptide RT. The 3 scans averaged signal centred at this time is then compared with the full theoretical peptide isotopic pattern. This additional quantification is compared to the first one to generate a final result report. The convergence of these two quantification routines is used to improve the quantification confidence and identification coverage.

References

1 A, B. DIFFTAL : A label-free approach for absolute quantification of proteins in a complex mixture. *presented at the annual meeting "6ème Journée de Spectrométrie de Masse en Midi-Pyrénées", Toulouse, France, 13 December 2011* (2011).
